# A Bibliometric Analysis of Neuroscience Tools Use in Construction Health and Safety Management

**DOI:** 10.3390/s23239522

**Published:** 2023-11-30

**Authors:** Zhikun Ding, Zhaoyang Xiong, Yewei Ouyang

**Affiliations:** 1 Key Laboratory of Coastal Urban Resilient Infrastructures (Shenzhen University), Ministry of Education, Shenzhen 518060, China; 2 Guangdong Laboratory of Artificial Intelligence and Digital Economy (SZ), Shenzhen 518060, China; 3 Sino-Australia Joint Research Center in BIM and Smart Construction, Shenzhen University, Shenzhen 518060, China; 4 Shenzhen Key Laboratory of Green, Efficient and Intelligent Construction of Underground Metro Station, Shenzhen University, Shenzhen 518060, China; 5 Department of Architecture and Civil Engineering, City University of Hong Kong, Kowloon, Hong Kong, China

**Keywords:** construction health and safety management (CHSM), neuroscience, bibliometric analysis, hazard recognition, fatigue monitoring

## Abstract

Despite longstanding traditional construction health and safety management (CHSM) methods, the construction industry continues to face persistent challenges in this field. Neuroscience tools offer potential advantages in addressing these safety and health issues by providing objective data to indicate subjects’ cognition and behavior. The application of neuroscience tools in the CHSM has received much attention in the construction research community, but comprehensive statistics on the application of neuroscience tools to CHSM is lacking to provide insights for the later scholars. Therefore, this study applied bibliometric analysis to examine the current state of neuroscience tools use in CHSM. The development phases; the most productive journals, regions, and institutions; influential scholars and articles; author collaboration; reference co-citation; and application domains of the tools were identified. It revealed four application domains: monitoring the safety status of construction workers, enhancing the construction hazard recognition ability, reducing work-related musculoskeletal disorders of construction workers, and integrating neuroscience tools with artificial intelligence techniques in enhancing occupational safety and health, where magnetoencephalography (EMG), electroencephalography (EEG), eye-tracking, and electrodermal activity (EDA) are four predominant neuroscience tools. It also shows a growing interest in integrating the neuroscience tools with artificial intelligence techniques to address the safety and health issues. In addition, future studies are suggested to facilitate the applications of these tools in construction workplaces by narrowing the gaps between experimental settings and real situations, enhancing the quality of data collected by neuroscience tools and performance of data processing algorithms, and overcoming user resistance in tools adoption.

## 1. Introduction

The construction industry ranks among today’s most hazardous sectors [1]. Consequently, health and safety on construction sites have emerged as a topic of primary concern for the global construction industry [2]. According to a report by the International Labor Organization, about one in six fatal work accidents occur in the construction industry [3]. Additionally, the incidence of non-fatal occupational injuries and illnesses in the construction sector is 30% higher than in other industries [4]. Despite the numerous safety and health regulations in the construction industry, construction accidents and occupational diseases remain high compared to other occupations [5]. This can be attributed to the inherently hazardous, dynamic, and often strenuous conditions under which construction work is performed [6]. The repercussions of these hazards not only affect the individual workers but also have broader implications for project timelines, costs, and overall industry reputation [7]. Over the years, traditional safety training programs, on-site safety inspections, and the establishment of safety policies and standards have been implemented to mitigate these risks; however, more innovations in prevention strategies and measures are needed, as the industry is saturated with safety and health management [8].

Potential solutions for public safety and health issues on construction sites have primarily focused on management strategies, education, regulations, and medical monitoring. However, these methods often rely on subjective, self-reported questionnaires prone to biases and memory distortions [9]. Ringen noted that data standardization allows for more accurate monitoring of safety and health issues [10]. In this context, neuroscience tools offer a real-time, objective data framework that can help overcome these limitations by providing standardized monitoring grounded in empirical measurements. Neuroscience tools can measure not only stimulus-induced physiological signals but also real-time brain activity signals [11].

The use of neuroscience tools on construction workers in construction sites also has great potential to improve workers’ safety and health. Construction sites are complex and involve hazardous environments. Traditional safety and health management approaches that rely on manual monitoring, such as safety inspections, require a large number of safety staff [12]. Also, the manual inspection does not allow for continuous and real-time monitoring, and it could only know the human behaviors. What is worse, manual inspections may be subjective and prone to errors. The application of neuroscience tools would allow for continuously and real-time monitoring of workers, and it also provides objective data [13]. Moreover, these tools also provide psychological data to indicate workers’ cognitive states, which provide warnings of their unsafe status in advance.

Neuroscience tools can be broadly categorized into “non-invasive brain measurement techniques” and “peripheral physiological measurement techniques”. The brain measurement techniques include Electroencephalography (EEG) for measuring changes in brain potential activity, Functional Magnetic Resonance Imaging (fMRI) for measuring changes in blood flow in the brain, Functional Near Infrared Spectroscopy (fNIRS) for measuring blood oxygen levels in the brain, and Magnetoencephalography (MEG) for measuring the magnetic field generated by neuron discharges in the brain. The peripheral physiological measurement techniques include Electrocardiogram (ECG) for measuring the electrical activity of the heart, Photoplethysmography (PPG) for measuring blood volume changes, Electromyogram (EMG) for measuring muscle electrical activity, Electrodermal Activity (EDA) for measuring changes in skin conductivity, and eye-tracking for measuring eye position and movement. These tools provide unique insights into the intricacies of human cognition and physiology by enabling the real-time monitoring of workers’ mental states. For instance, EEG can track changes in brain waves to monitor and assess the cognitive status of construction workers, such as attention and vigilance [14], fatigue [15], stress [16], as well as the effects of the work environment on subjects’ emotion [17]; also, EDA monitors sweat gland activity to identify increases in psychological stress [18]. Moreover, eye-tracking allows continuous assessment of workers’ visual attention allocation and hazard identification [19,20,21,22].

Several reviews on CHSM based on neuroscience tools have been conducted and published [13,15,23,24,25,26]. Among existing reviews, the adoption of EEG has received the most interest. Cheng et al. [13] conducted a critical review to examine the applications of EEG in measuring and computing construction workers’ cognitive statuses; Saedi et al. [24] conducted a systematic review for revealing the potential applications of mobile EEG on construction sites and investigating the contribution of this technology to workers’ wellbeing and safety; Zhang et al. [23] summarized the existing studies that have involved EEG and construction safety thorough a systematic review. Regarding a broader range of neuroscience tools, a critical review from Ahn et al. [25] examined the application domains of wearable sensing technology in construction safety and health; some of the neuroscience tools, including EEG, ECG, PPG, eye-tracking, and EMG, were covered in the wearable sensing technology, but there was a lack of discussion on the neuroimaging tools fNIRS and fMRI which have begun to be used in construction safety in recent years. Wang et al. [11] applied a bibliometric analysis to analyze the current research of applying neuroscience tools in the whole building life cycle. They pointed out that construction safety and occupational health are two main application domains, but they failed to provide further discussion of specific applications of these tools in the safety and health management. A comprehensive grasp in CHSM domain incorporating the utilization of neuroscience tools is lacking. This study hence proposed to have a comprehensive and holistic bibliometric analysis of CHSM research that incorporates the utilization of neuroscience tools.

The bibliometric analysis was selected because its quantitative analysis can avoid subjectivity and lacking reproducibility [27]. It can provide a more in-depth understanding of the facets embodying diverse research emphases over the years, as well as the relationships between them [28]. It is also capable of unraveling and mapping the complexity of knowledge accumulation and evolution in established fields by handling large amounts of unstructured data [29].

This analysis encompasses various dimensions, including performance analysis, science mapping, and content analysis. This study covers the statistical analysis of publication counts, geographical distribution, authorship patterns, and keyword trends during the period from 2001 to 2023. The objectives of this study include: (1) to analyze influential journals, keywords, scholars, and articles in the field of neuroscience tools use in CHSM; (2) to analyze existing mainstream research topics in the field of neuroscience tools use in CHSM; (3) to discuss the limitations or gaps and to propose a research framework to guide future scholarship and research efforts.

## 2. Materials and Methods

### 2.1. Research Framework

**Figure 1:**
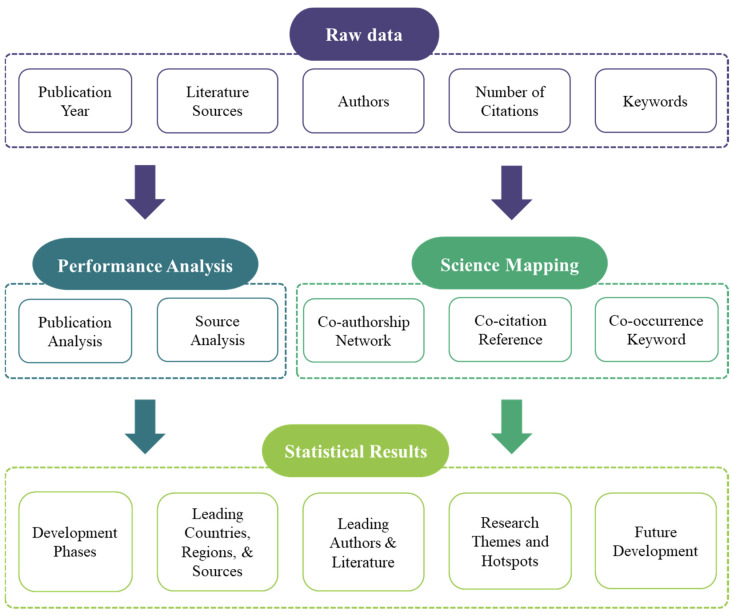
Research framework.

The framework of the bibliometric analysis in this study is shown in Figure 1. First, basic information of the related literature, including the publication year, publication sources (journal, country, and institution), authors, number of citations, and keywords, were extracted for further analysis. 

Then, the two kinds of bibliometric techniques, performance analysis, and scientific mapping, were applied [29]. The performance analysis is a type of descriptive analysis, which specifies the characteristics of bibliometric studies [30]. It uses indicators such as the number of publications, citations, and the H-index to measure the productivity and impact of countries or regions, sources, and authors. It was used for analyzing the publication years and sources in this study. The science mapping was used to visualize the connections between elements like authors, publications, sources, and countries to enable an enhanced understanding of research landscapes and dynamics within a research field [31]. Specifically, it was applied for co-authorship network, which indicates the leading collaboration communities; co-citation reference analysis, which reveals the links between pivotal publications; and co-occurrence keywords analysis, which shows relationships between important concepts or topics. 

Finally, based on the performance analysis and science mapping, the development phases, leading countries or regions, institutions and main journals, leading authors and literature, research themes and hotspots, limitations, and future prospects of this research field were obtained.

### 2.2. Publications Search and Selection

**Figure 2:**
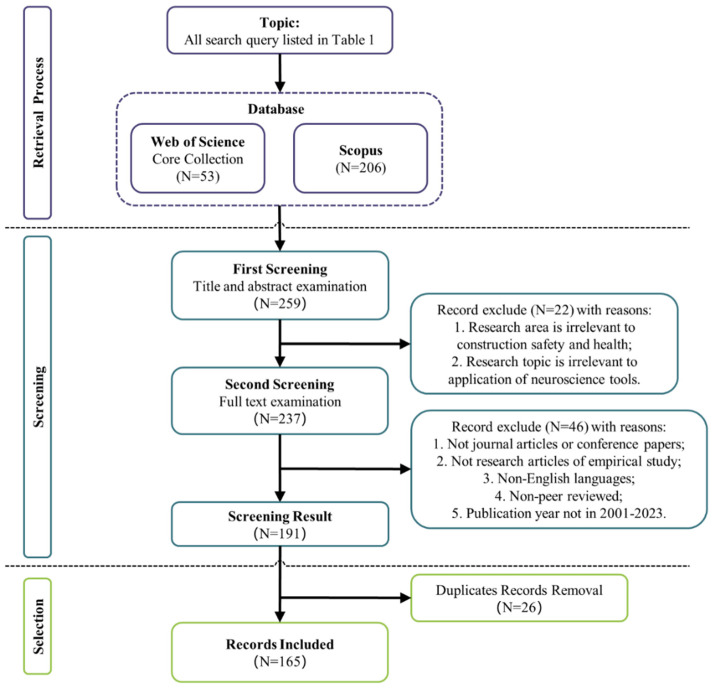
PRISMA flowchart for selecting publications for the current study.

In order to obtain a robust research process, this study strictly adheres to the Preferred Reporting Items for Systematic Reviews and Meta-Analyses (PRISMA) guidelines [32,33]. The PRISMA framework, depicted in Figure 2, is organized into three pivotal stages: (i) Retrieval Process, (ii) Screening—comprising both First and Second Screening phases, and (iii) Final Selection. This structured approach facilitates a nuanced exploration, ensuring the review process remains comprehensive and discerning.

#### 2.2.1. Databases Accessed

In the Retrieval Process, a systematic search strategy was implemented. Initially, the Web of Science, Scopus, and PubMed online databases were chosen as the primary data sources. Web of Science is known for its comprehensive coverage of journals across various disciplines, making it suitable for the multidisciplinary nature of neuroscience in CHSM. Meanwhile, Scopus is widely used among researchers in engineering and management [34]. PubMed is a comprehensive database that covers biomedical and life sciences literature, including the latest cutting-edge achievements in neuroscience [35]. Integrating the above three online databases provides the necessary support for a comprehensive search of relevant literature at the intersection of CHSM and neuroscience.

#### 2.2.2. Search Strategy

This study explored the use of neuroscience tools in CHSM between 2001 and 2023. A combination of search queries inspired by prior reviews, outlined in Table 1, was employed to ensure that the most relevant and comprehensive research was obtained. The use of advanced search categories coupled with Boolean operators further refined the retrieval process.

#### 2.2.3. Include & Exclude Criteria

To ensure a rigorous and comprehensive exploration of literature regarding the application of neuroscience tools in CHSM, we implemented a stringent inclusion and exclusion protocol, depicted in Figure 2. The screening procedure was methodically divided into three stages to refine the extracted documents from our initial retrieval of 259 articles.

Stage 1: The first screening aimed at ensuring relevance. Only articles centered on CHSM, and those with a clear focus on the application of neuroscience tools, were remained. From this initial screening, 22 articles were considered irrelevant and discarded, and 237 articles were allowed into the second screening.

Stage 2: The second screening was dedicated to ensuring the quality and specificity of the chosen literature. Only empirical studies published as journal articles or conference papers and published between 2001 and 2023 were selected for inclusion in this review. Furthermore, to uphold research integrity, only peer-reviewed English publications were considered. This step excluded 46 articles, reducing the pool to 191.

Stage 3: Beyond the primary search mechanism, additional methods were incorporated: manual examination of reference lists from the shortlisted articles, citation tracking of seminal papers, and consultation with domain experts to validate the relevance and significance of our chosen literature. To prevent redundancy, 26 duplicate articles were removed. Ultimately, 165 articles were deemed fit for this study.

#### 2.2.4. Science Mapping

After completing data preprocessing involving synonym merging and eliminating irrelevant words, science maps were generated, including the co-authorship network of authors and countries (regions), co-citation network of reference, and co-occurrence network of keyword cluster, which could depict the integration and evolution of neuroscience tools in CHSM research. These science maps were obtained by standardizing author names and keywords to consolidate variants that refer to the same concepts first, then putting data from Scopus, Web of Science, and PubMed into the bibliometric analysis software. The software of bibliometric analysis applied in this study includes VOSViewer 1.6.19 and Bibliometrix 4.1.3. VOSviewer, developed by Leiden University in the Netherlands, is a professional tool for structuring and visualizing bibliometric networks. The layer-labeling structure of VOSviewer effectively showcases interactions within dense network nodes, making it ideal for examining intricate networks and handling vast data from extensive citations and keyword co-occurrences [36]. Bibliometrix is an open-source online visualization software based on RStudio [37]. In bibliometric analysis, Bibliometrix excels in statistical evaluations, indexing computations, and network assessments. It encompasses all essential tools for a comprehensive bibliometric analysis in line with scientific mapping protocols, enhancing the sophistication and reproducibility of such analyses [38].

## 3. Results and Analysis

### 3.1. Development Phases during 2001 to 2023

**Figure 3:**
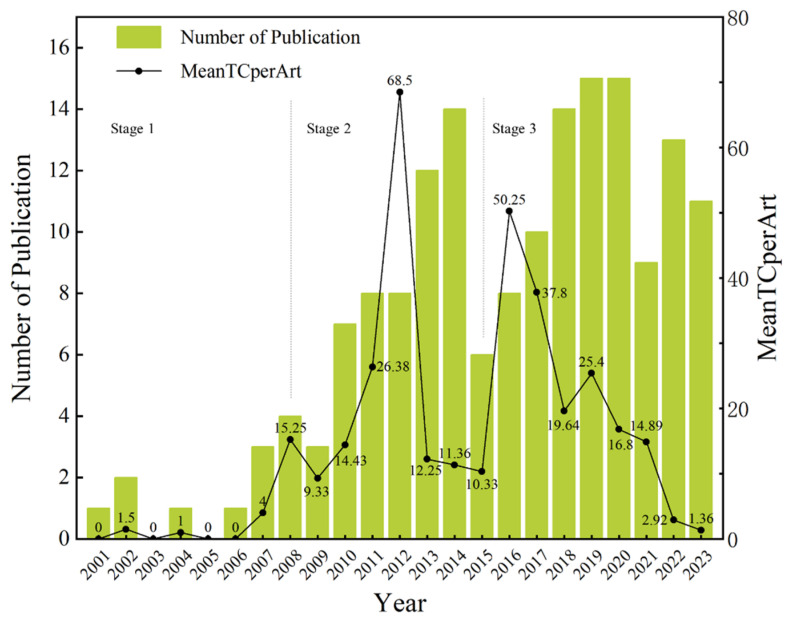
Annual publications from 2001 to 2023.

The evolution of a research field can be traced through the ups and downs of its publication history. As depicted in Figure 3, an examination of the literature from 2001 to 2023 revealed that the level of interest in this field is still increasing. Over these years, a total of 165 publications have been published. The exploration of the development trend in the application of neuroscience tools in CHSM revealed several phases.

Phase 1 (2001–2007): Nascent period. The initial phase of development spanned from 2001 to 2007. During this period, the field was in its infancy, with publication numbers remaining relatively low. No year within this phase saw more than two publications, signifying the research area’s early exploration and groundwork-laying stage.

Phase 2 (2008–2014): Rapid ascension. The research field experienced a remarkable surge in publication output in this period. Commencing in 2008, there was a 33% increase over the previous year, with four papers being introduced. Each year within this interval successively outdid the previous one, and this momentum did not wane. The number of publications reached a peak of 14 in 2014, representing a 467% increase compared to the number published in 2007. This surge could be attributed to the widespread adoption of EMG technology, which has established its utility in CHSM.

Phase 3 (2015–2023): Contemporary trends. From 2015 to 2023, the publication pattern stabilized, with the number of annual publications fluctuating between 8 and 15. There was a decline in 2015 followed by sustained growth, likely linked to the introduction of new technologies like EEG and eye-tracking in 2016. As the field matured, publications declined slightly to 9 in 2021 but rebounded to 13 in 2022, showing the resilience and continued interest in this research area. In 2023, 11 publications attested to steady development. Although promising, this field remains in its developing phase, with numerous opportunities arising from emerging evidence.

An additional aspect worth considering is the H-index, which indicates the impact and scholarly influence of the research in certain years. Similar to the annual publication data trend, this metric experienced significant oscillations over the years. A noteworthy peak was reached in 2019, with a count of nine. However, the subsequent two years have declined, suggesting varying levels of influence and recognition within the academic community. It may be because the latest research has not yet reached sufficient citations and needs more time to prove its value.

### 3.2. Publication Sources over the Years: Journals, Regions, and Institutions

#### 3.2.1. Journal Analysis

**Figure 4:**
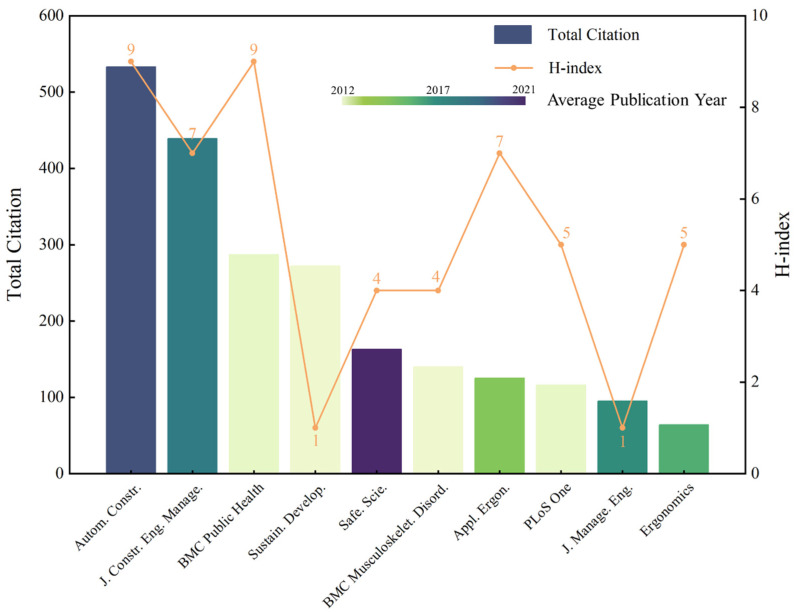
Source analysis.

This study presents a comprehensive view of source impact. As shown in Figure 4, the total citations are represented by columns indicating quantity. The H-index is illustrated with a dotted line, signifying quality. Different column colors denote the average publication year, serving as an indicator of recency. The top 10 sources in total citations were *Automation in Construction* (533 total citations), *Journal of Construction Engineering and Management* (439), *BMC Public Health* (287), *Sustainable Development* (272), *Safety Science* (163), *BMC Musculoskeletal Disorders* (140), *Applied Ergonomics* (125), *PLoS One* (116), *Journal of Management in Engineering* (95), and *Resources Policy* (89). When observing the broader picture, *Automation in Construction* excelled in all metrics, with the highest total citations (533) and H-index (11), which reveals its significant impact and activity in the field. To clarify the main topics related to the field of interest within *Automation in Construction*, a detailed analysis was undertaken. Specifically, titles, abstracts, and keywords of all relevant articles published in the journal were reviewed. The most frequent 20 terms were extracted to discern the main topics. The textual analysis revealed that one of the main topics in *Automation in Construction* is related to the fatigue, mental or cognitive safety, and health of construction workers.

Similarly, the *Journal of Construction Engineering and Management* has made substantial contributions, particularly in hazard recognition and occupational health, with a strong inclination towards leveraging computer vision technologies. As for *Safety Science*, it emerged as an evolving source with its debut publication in 2016 and pronounced interest in safety training and management, which points towards prospective trends in research. Conversely, *BMC Musculoskeletal Disorders*, which started in 2007, has diminished in recent activity. In addition, *BMC Public Health* boasts a higher H-index (9) in comparison to *Journal of Construction Engineering and Management* (7), even though its total citations (287) are dwarfed by the latter’s 439. This discrepancy suggests that a higher H-index does not necessarily correspond with a higher citation count.

A domain-centric analysis revealed that construction engineering journals, notably *Automation in Construction*, *Journal of Construction Engineering and Management*, and *Journal of Management in Engineering*, outperformed non-construction journals across various metrics. Specifically, construction engineering sources averaged a total citation (TC) of 392.67, compared to 166.71 for non-construction engineering sources. In terms of the number of publications (NP), construction engineering sources averaged 6.67, while their non-construction counterparts averaged 5.14. Additionally, the average publication year for construction engineering journals was more recent, standing at 2018, compared to 2014 for non-construction engineering journals.

This superior performance of construction journals can be attributed to their specialized focus on worker health and safety and the direct relevance of their research findings. As the industry evolved and integrated advanced neuroscience tools, construction scholars began to leverage these tools, building on insights from earlier non-construction research. Furthermore, the higher citation count for construction-centric journals underscores their influence within the research community. Despite the slightly subdued performance of non-construction journals, their contributions highlight the interdisciplinary nature of the research. As the field progresses, construction journals will likely remain central, with interdisciplinary collaborations continuing to offer fresh methodologies and perspectives.

#### 3.2.2. Country (Region) and Institution Analysis

**Figure 5:**
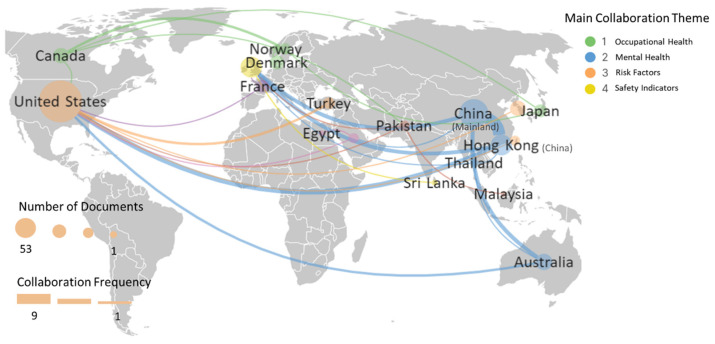
Co-authorships network of the top 30 countries (region) based on publication numbers.

Figure 5 depicts the distribution of publications and the collaborating network between countries or regions worldwide. This distribution has been obtained statistically from the institutional address or affiliation of the corresponding author. These countries (regions) were then grouped into clusters based on a separate analysis of international collaborations by country co-authorships network in VOSviewer. The top 20 frequent terms for each cluster were analyzed to identify the primary research theme of each cluster. The green cluster, representing “Occupational Health”, with a main representation from countries such as Canada, Norway, Pakistan, and Japan, emphasizes the significance of ensuring occupational health of workers in construction environments. Representative works in this cluster include the studies of [39,40,41,42,43]. The blue cluster, signifying “Mental Health”, encompasses countries or regions like China, Hong Kong, and Australia, focusing on mental health nuances within CHSM. Representative works in this cluster include the studies of [19,44,45,46,47]. The orange cluster, centered on “Risk Factors”, led by countries and regions like the United States, South Korea, Taiwan, and Turkey, illustrates the identification of risk factors in construction processes and environments. Representative works in this cluster include the studies of [48,49,50,51]. The yellow cluster, dedicated to “Safety Indicators”, primarily involves Denmark and Sri Lanka. This theme emphasizes the diverse safety indicators essential for evaluating and ensuring safety standards, with a prominent contribution being [52].

The size of the bubble in Figure 5 provides insights into the volume of published documents, and the number and thickness of the connecting lines offer a view of the frequency and intensity of collaboration. The United States had the highest number of publications, with 53 documents and 1283 citations. It was followed by China (24 publications, 659 citations), Hong Kong (13 publications, 580 citations), the United Kingdom (13 publications, 113 citations), Australia (8 publications, 125 citations), Canada (7 publications, 105 citations), South Korea (7 publications, 94 citations), Denmark (6 publications, 125 citations), France (5 publications, 87 citations), Japan (5 publications, 23 citations), Sweden (4 publications, 70 citations), and Turkey (4 publications, 43 citations). It shows that the United States maintains a dominant position in traditional metrics of scholarly collaborations; countries (regions) like China, Hong Kong, and especially Australia demonstrate an edge in the recency of academic engagements according to the average publish year. This suggests that these regions have been particularly active in contemporary scholarly endeavors, reflecting a strategic emphasis on current research topics and methodologies. The United States also shows a clear lead in terms of partnership breadth, indicating its significant influence and activity in the world, reflected in the most significant number of color types through the U.S. connection to other countries. China also demonstrates high regional cooperation activity, especially the strongest level of collaboration strength with the Hong Kong Special Administrative Region, which can be seen as the thickest link in all connections.

From the perspective of the institute shown in Table 2, the City University of Hong Kong and Tsinghua University emerged as leading institutions, each boasting four publications. The City University of Hong Kong demonstrated significant academic influence, with its works being cited 295 times. Other institutions included the EMGO+ Institute for Health and Care Research, Purdue University, and the University of Florida, each with three publications. Whereas Purdue University’s works garnered 43 citations, the University of Florida’s research was cited 31 times.

### 3.3. Influential Scholars and Author Collaboration

#### 3.3.1. Influential Scholars

Table 3 highlights the scholarly contributions of the top 15 authors. Considering the publication count, total citations, H-index, and average publication year, it indicates that Van Der Molen, Henk F and Boschman, Julitta S. stand out as trailblazing researchers in the arena of occupational safety and health for construction workers using various physiological tools. Their seminal works in the early 2010s set the foundation for subsequent research in this domain [53,54]. Li Heng stands out as a luminary in the topic of “Construction Informatics”, “Construction Engineering and Management”, and “Construction Health and Safety”. He has the highest citation count of 273, which proves that his scholarship has a broad impact. Moreover, his five publications signal a constant and meaningful engagement with the field. Chen, Jiayu and Esmaeili, and Behzad both keep an H-index of 5. The former has made outstanding contributions mainly in “Human-centric Mental Sensing”, while the latter specializes in discussing “Construction Safety”, “Risk Management” and “Decision Making”. On the contrary, while possessing a high citation count of 272, Berardi Umberto attributes most of this recognition to singular influential work in “Sustainability and Building Science”, which becomes evident with his H-index of 1. Choi Byungjoo, and Jebelli Houtan both displayed a recent surge in research contributions centered around 2020. Choi’s research gravitates toward “Smart Construction and Construction Safety”, while Jebelli focuses on “Construction Robotics” and “Wearable Technologies”. Other authors have also covered a wide range of research topics, including “Deep Learning”, “Signal Processing”, “Artificial Intelligence in Building Management”, “Cognitive Neural Engineering”, “Brain-Computer Interfaces”, “Occupational Health”, and “Musculoskeletal Disorders of Construction workers”.

#### 3.3.2. Author Collaboration

**Figure 6:**
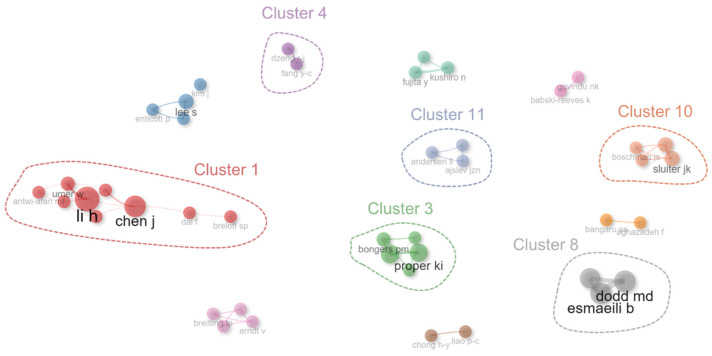
Co-authorships network of author collaboration.

Figure 6 shows the results of an analysis of the authors collaborative network within CHSM. A total of 12 clusters representing distinct research communities were discerned from the collaboration data. Of these, the six core clusters with the highest total citations were selected for further analysis. The first is Cluster 1, mainly composed of Chen Jiayu, Dai Fei, and Li Heng et al. This cluster had the highest academic impact, amassing a remarkable 1291 citations. The highly cited publications included studies of [14,45,55]. These studies focused on the mental and physical fatigue of construction workers, emphasizing the utilization of EEG in monitoring construction workers’ mental states. Cluster 4 and Cluster 8 are two thematically similar clusters, both focusing on risk perception, situation awareness, and hazard identification for construction workers based on eye-tracking [20,21]. The former consists of Dzeng, Ren-Jye, Fang, and Yi-Cho et al., and the latter is mainly formed by Hasanzadeh Sogand, Esmaeili Behzad, and Dodd Michael D. Clusters 3, 10, and 11 highlight research on interventions to improve the physical and mental health of construction workers. Cluster 3, illuminated by Proper Karin I, Van Der Beek Allard J, and Groeneveld Iris F, focuses on the importance of lifestyle modifications to address cardiovascular disease risks, with emphasis on both short and long-term impacts [56]. Cluster 10, represented by Sluiter Judith K, Van Der Molen Henk F, and Boschman Julitta S., examines musculoskeletal disorders among construction workers specifically [54]. Cluster 11 relates to participatory ergonomics interventions for preventing musculoskeletal disorders, with key works by Brandt Mikkel, Ajslev Jeppe Z.N., and Andersen Lars L. [42,43]. Together, these three clusters demonstrate a research emphasis on exploring various health interventions, including lifestyle changes, ergonomic adjustments, and targeted training, to improve the physical and mental health of construction workers. The collaboration data have revealed key research communities in construction research. This analysis not only identifies the major players and their contributions but also offers a roadmap for future research directions in the field.

### 3.4. Highly Cited Articles and Reference Co-Citation Analysis

#### 3.4.1. Top 10 Most-Cited Papers

Table 4 represents the top 10 most-cited papers in CHSM, published from 2012 to 2019, which exhibit a substantial impact on the academic and practical community. The most cited paper, “Using eye-tracker to compare search patterns between experienced and novice workers for site hazard identification” by Dzeng et al. [20], published in *Safety Science* in 2016, has the highest number of citations, 111. This article examines the impact of experience on hazard identification in digital construction sites using eye-tracking, highlighting the difference between speed and accuracy. At the same time, Chen et al. [45] obtained significant attention with 100 citations. The article of Wang et al. [14] published in *Automation in Construction* obtained 98 citations. Following references are [54] (total cited by 97), [21] (total cited by 95), [55] (total cited by 91), [22] (total cited by 87), [57] (total cited by 70), [58] (total cited by 66), [59] (total cited by 63). Interestingly, six of the ten most cited articles used eye-tracking, which has been well received by researchers. All the articles published in highly reputed journals with high impact factors influence researchers to encourage publishing articles on this topic in these highly reputed journals and develop future research areas. 

#### 3.4.2. Reference Co-Citation Analysis

Co-citation refers to the concurrent citation of two documents by a third distinct paper. The frequency with which two documents are co-cited indicates the thematic relationship between their content [60]. Such co-citation patterns can reveal “classic” or foundational publications in a research field. Importantly, these patterns are dynamic and may evolve over time, reflecting shifts in the field’s focus or paradigms. This temporal variability in co-citation not only provides valuable insights into the development of the field but also aids in identifying paradigmatic changes [37].

**Figure 7:**
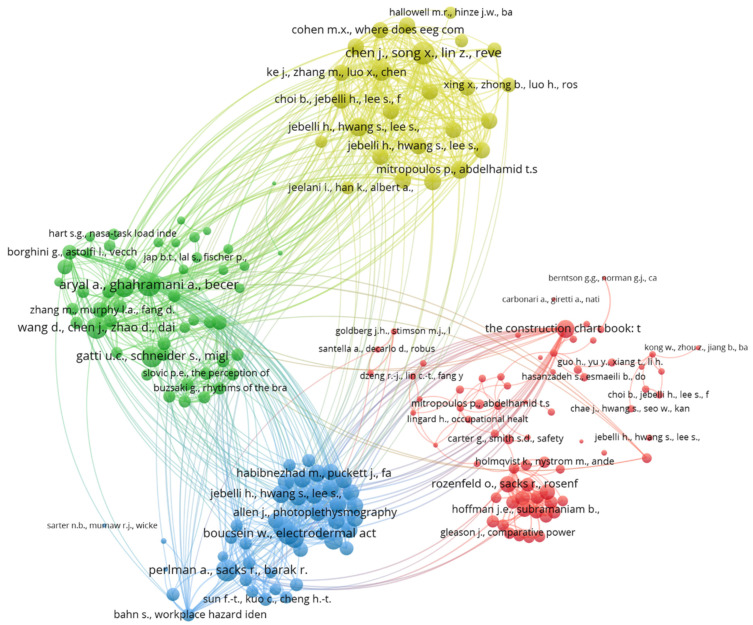
Reference co-citation.

The reference co-citation network of neuroscience tools used in CHSM is shown in Figure 7. The size of the bubbles represents the number of standardized citations received by the article, and the thickness of the lines represents the strength of the co-citation relationship. Links and proximity between two articles identify the co-citation relationship between them. The color of the bubbles indicates the cluster associated with the article. Each bubble is labeled with the first author of the article and the year of publication. A total of four clusters were generated from the VOSviewer co-citation analysis. Cluster 1 (red) primarily concentrates on site safety and risk identification, especially the physical safety of workers. Key topics include hazard recognition and identification [61], the use of wearable tracking devices [62,63], and eye-tracking technologies [19]. Cluster 1 also emphasizes fatigue management [64,65] and task analysis [66,67]. These works often employ various models and monitoring techniques to evaluate workers’ safety in real-world construction sites. Cluster 2 (green) focuses on exploring the psychological dimensions of workers’ safety in the construction industry and safety education and training. The papers delve into mental workload [68], cognitive function [69], and brain activity, often utilizing EEG analysis [70]. Taxonomy and educational objectives related to safety are also featured prominently [71,72]. Cluster 3 (blue) revolves around the effects of physiological stress recognition, responses, and environmental factors [73] on workers in the construction industry. Cluster 3 also explores the influence of occupational factors such as high altitudes [74] and physical workload [75]. Cluster 4 (yellow) primarily engages with data analysis methodologies, particularly those related to EEG dynamics. Key tools include open-source toolboxes like EEGLAB [76], and key methods include independent component analysis in single-trial EEG studies.

### 3.5. Application Domains of Neuroscience Tools in CHSM

**Figure 8:**
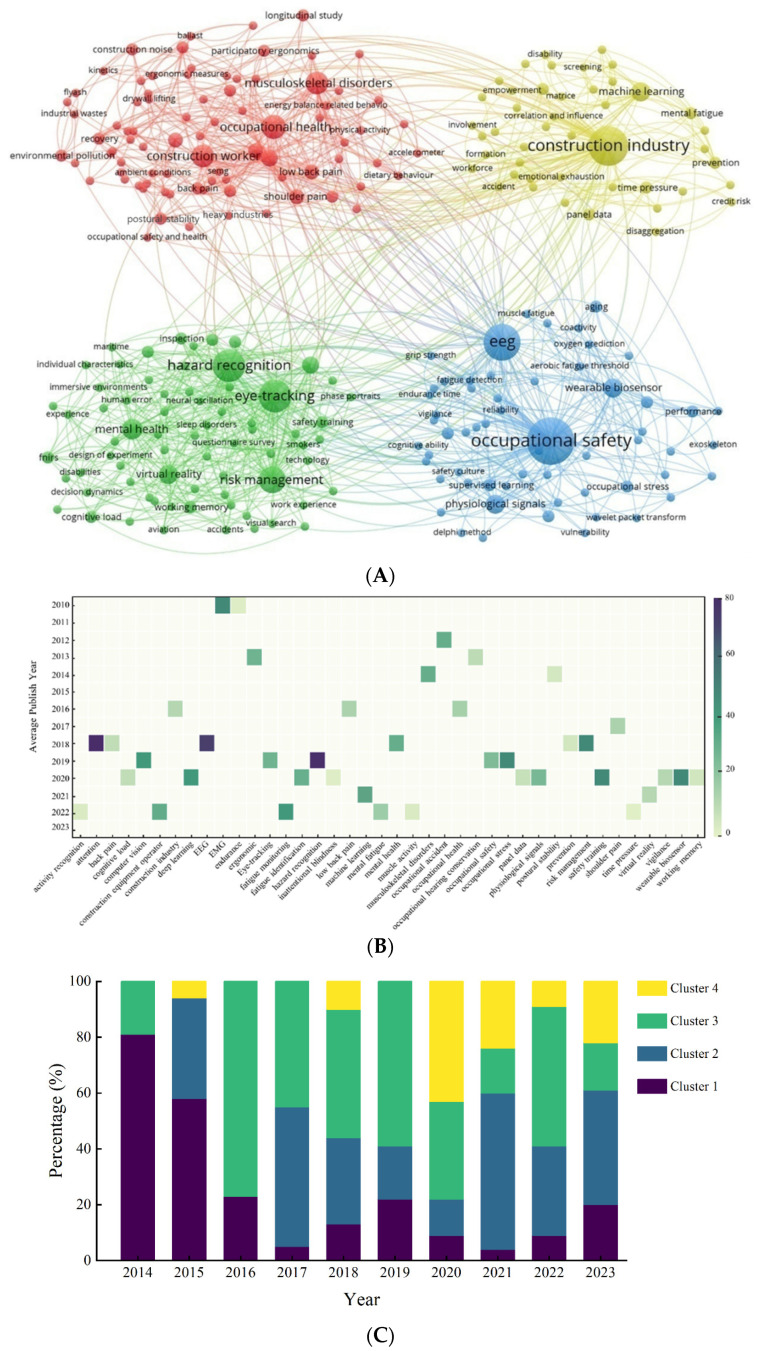
Results of application domains of neuroscience tools in CHSM and its trend. (**A**). Co-occurrence keywords network. (**B**) Top 40 keywords by average citations. (**C**). Percentage distribution of the average citation of keywords by cluster over the years.

Keywords analysis was applied to extract the application domains of neuroscience tools within the CHSM. The results of keyword co-occurrence network are shown in Figure 8A. Four clusters were identified which are distinguished by color, and the node size corresponds to the frequency of keyword appearance. In addition, the degree of centrality metric was applied to each keyword across the network to identify core keywords. Core keywords were extracted for each of the four clusters, with the results shown in Table 5 (the number following each keyword indicates its degree of centrality within the network). Keywords with higher centrality are more interconnected and cover diverse research topics, signifying core issues and directions [29]. 

To conclude, it indicates that there are four application domains which can be concluded as: monitoring workers’ safety status, enhancing workers’ hazard identification ability, reducing work-related muscle skeleton disorders of construction workers, and integrating with artificial intelligence (AI) to address the safety and health issues. Furthermore, the evolution of research trends over the period was analyzed by examining the keyword trends (Figure 8B, showing the top 40 keywords based on average citations) and the dynamic shifts in research focus (Figure 8C, showing the percentage of research of the four application domains). Specific details of the four application domains are clarified below. 

#### 3.5.1. Monitoring Workers’ Safety Status

A typical application is to monitor workers based on physiological measurement techniques, as shown in the blue cluster (Cluster 1) of Figure 8A. It focused on applying EEG and other wearable biosensors to measure workers’ cognitive status which is associated to their safety, such as attention and fatigue, which ultimately could enable personalized real-time safety interventions tailored to individual worker states. For instance, many studies [77,78,79] have focused on identifying stress in construction workers by capturing the brain activity of construction workers using EEG signals. Ke et al. [80] examined the effects of various noise conditions on task performance and cognitive performance via a portable EEG device to draw attention to the cognitive characteristics of distraction and the quantitative detection method. Hasanzadeh et al. [81] measured real-time situational awareness of construction workers in different scenarios in a real construction site using a mobile eye-tracking device. They found that the workers’ situational awareness and visual attention allocation vary significantly depending on the workload of the scene, the state of the region of interest, and the worker’s experience level. Chen et al. [45] discussed the problem of “inattentional blindness” in construction using the analogy of the “invisible Gorilla” experiment, which shows that people can miss obvious and unexpected events when they are focused on a specific task. 

In addition, monitoring workers’ safety status illustrates the industry’s aspiration for taking proactive interventions to enhance construction safety. This application domain has also been demonstrated to be the research focus areas of construction safety, which can be indicated by the high occurrence resilience of terms related to “EEG”, “attention”, and “fatigue monitoring and identification “in recent years in Figure 8B. As shown in Figure 8C, it is worth noting that although the topics associated with Cluster 1 have dominated the discourse historically, there is a discernible decline in recent years that suggests evolving research interests or the maturation of these technologies. 

#### 3.5.2. Enhancing Workers’ Hazard Identification Ability

The research in Cluster 2 (green) in Figure 8A aims to enhance construction workers’ hazard identification ability. The cluster foreshadows the potential of emerging technologies like VR and biosensors to enable the development of highly personalized and adaptive safety training systems for targeted enhancement of hazard identification. Eye-tracking has been a primary tool in this domain. Eye-tracking is often used to analysis the characteristics and influence factors of workers’ visual attention [22]. Hasanzadeh et al. [21] used remote eye-tracking technology to explore the effects of safety knowledge (including training, work experience, and injury experience) on construction workers’ attention allocation and hazard detection. Li et al. [70] utilized eye-tracking and virtual reality to examine visual attention and hazard recognition performance. They found that mental fatigue induced by prolonged monotonous operations can impair hazard detection and increase the risk of accidents.

In addition, developing safety training approach integration with virtual technology has also been a research focus. Active safety training for construction workers is imperative to improving their risk alertness, attention levels, and risk perception [82]. Safety training leveraged virtual and mixed-reality technologies to provide immersive and engaging experiences. Fujita et al. [83] described a training tool for video risk identification using an eye-tracking device, and by comparing the attention allocation of veterans and novices, it was found that the veterans’ risk identification is based on meta- and domain knowledge. Buekrue et al. [84] developed an immersive VR safety training system in the construction industry with motion tracking, eye-tracking, realistic tools, and destructible elements to generate objective trainee data for personalized feedback and improved safety awareness. A virtual avatar training platform was developed by Shayesteh et al. [18], using physiological signals and deep learning to evaluate cognitive load and safety performance during human-robot collaboration tasks for improved construction safety training. Regarding the research trend of this application domain, the results of percentage distribution over the years (Figure 8C) shows that it has maintained a certain percentage. It indicates the ongoing importance of this topic for research in the construction field.

#### 3.5.3. Reducing Work-Related Muscle Skeleton Disorders of Construction Workers

Cluster 3 (red) in Figure 8A focuses on mitigating occupational health risks and promoting the overall wellbeing of construction workers. This area of research has dominated all these years (Figure 8C), showing the importance given to this health issue. The neuroscience tools were applied to recognize risk factors that may cause workers’ muscle skeleton disorders and evaluate the risk levels. Activities such as prolonged standing, bending, lifting heavy objects, and adopting awkward postures [85,86,87] were identified as significant contributors to prevalent health issues among workers. These behaviors were directly linked to key concerns such as musculoskeletal disorders, low back pain, and fatigue. In the quest to understand and address these health challenges, Electromyography (EMG) has emerged as an invaluable tool, shedding light on muscle endurance, physical fatigue, and the risks associated with musculoskeletal disorders. Merkus et al. [39] integrated accelerometers, surface Electromyography (sEMG), and Electrocardiography (ECG) to probe the physical demands and strain experienced by older construction workers. Seo et al. [88] utilized EMG to evaluate chronic muscle fatigue—one of the principal contributors to musculoskeletal disorders—in areas like the arms, lower back, and neck. Mudiyanselage et al. [89] introduced an automated ergonomic risk assessment method for manual tasks, leveraging sEMG wearable sensors and machine learning. However, the reliability of EMG data can be compromised by factors such as human errors, subject non-compliance, inherent signal variability, physiological states, and environmental conditions. Addressing this, some researchers have proposed fusing multiple incomplete datasets for data compensation [90]. It is worthy to recognize that the vulnerability to work-related musculoskeletal disorders in construction workers is not merely a consequence of physical exertion; psychological and social factors also contribute substantially to this tendency [91]. Therefore, the necessity of advanced ergonomic research and interventions, which integrate technological, psychological, and social factors, is evident. 

#### 3.5.4. Integrating with Artificial Intelligence (AI) to Address the Safety and Health Issues

The last Cluster 4 (yellow) emphasizes integrating the neuroscience tools with artificial intelligence (AI) technology, especially “deep learning”, “machine learning”, and “computer vision”, to enhance safety within the construction sector. AI offers innovative solutions for predicting and addressing safety concerns. For instance, the integration of AI technologies and wearable sensors has been instrumental in continuously monitoring workers’ mental states, underscoring the equal importance of mental and physical well-being. Mehmood et al. [44] successfully utilized a fusion of data sources, including electroencephalography and video signals, to classify mental fatigue with notable accuracy. Yu et al. [55] ventured beyond traditional methods, employing computer vision for comprehensive fatigue assessments across varied construction scenarios. Similarly, Lee et al. [51] harnessed machine learning and wearable sensors to assess the perceived risk levels of construction workers with an impressive 81.2% accuracy rate. Furthermore, Antwi-Afari et al. [92] employed deep learning, combined with wearable insole data, to proactively identify and categorize awkward working postures, aiming to combat musculoskeletal disorders in construction workers. 

There are still many challenges remaining in AI technologies, particularly deep learning, machine learning, and computer vision, in offering promising enhancements in construction safety in Cluster 4. The reliability of AI predictions can be compromised by incomplete or biased training data. Additionally, the complex nature of construction sites, characterized by ever-changing conditions and diverse worker behaviors, necessitates continuous learning and adaptation by these AI systems. However, the results in Figure 8B show that there is a growing interest in computational tools, particularly “machine learning” and “deep learning”, signaling the increasing role of data-driven approaches in enhancing construction safety. Moreover, Figure 8C also indicates the burgeoning interest in this research topic. This rise denotes the potential of harnessing advanced algorithms for real-time monitoring and predictive analytics, leveraging the vast troves of data these technologies can capture to achieve an intelligent CHSM.

### 3.6. Application Topics of the Primary Neuroscience Tools in CHSM

**Figure 9:**
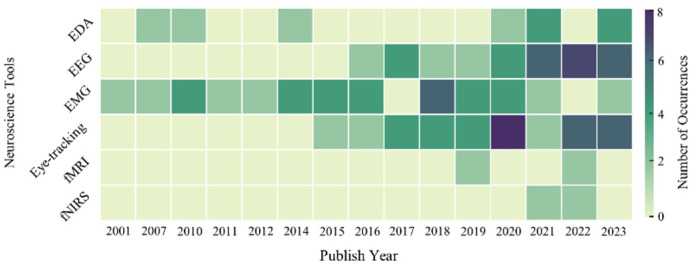
Neuroscience tools usage by year.

A thorough examination of neuroscience tools was conducted, emphasizing their role in CHSM. This chapter delves deeper into the distribution of these tools via keyword co-occurrence networks of each tool. Firstly, the neuroscience tools usage over time was examined, with the results shown in Figure 9. Among the tools, EMG consistently emerges as a wide range of applications, with its frequent applications extending from 2001 to 2016, illustrating its vital role in construction safety research. In contrast, fMRI and fNIRS, though integral, exhibit intermittent usage patterns, suggesting their specific applications or potential emerging relevance in certain periods. Another observation is the marked uptick in the adoption of eye-tracking technology from 2015, reaching a zenith in 2020. This surge could be attributed to technological advances and popularity, or an amplified research focus on domains where eye-tracking provides pivotal insights. Regarding EEG applications, its consistent use, especially from 2016 to 2021, demonstrates the effectiveness and stability of this technology in capturing neurophysiological data. In terms of EDA, after a phase of subdued interest, it has been re-emphasized in 2020 and 2021, indicating either its renewed significance or innovative applications in contemporary research. To conclude, EEG, eye-tracking, and EMG are the primary neuroscience tools used in related issues of CHSM. Therefore, the application of these four tools in the research area is further explained.

#### 3.6.1. EMG

**Figure 10:**
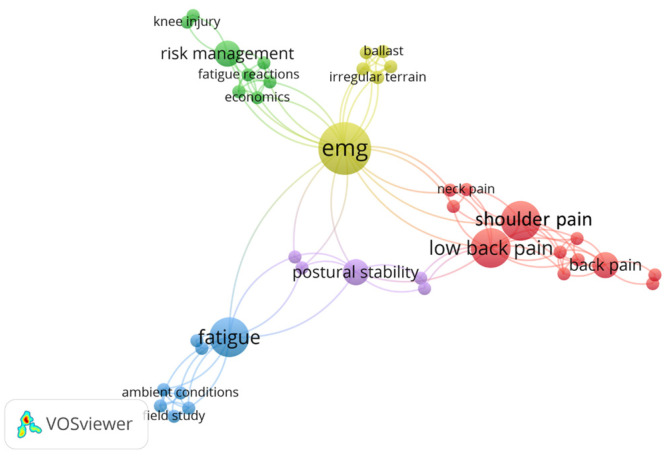
Keywords co-occurrence network of EMG in CHSM.

Figure 10 presents a co-word network of EMG applications in construction safety. The application of EMG in CHSM focuses on the physical strains, particularly those arising from prolonged or repetitive tasks, like “lifting” or maintaining certain “postures”. By capturing the electrical activity of muscles, EMG offers a direct window into muscle exertion and potential fatigue, which becomes particularly crucial when assessing the risks associated with “musculoskeletal disorders”. Dutta et al. [93] highlighted the significant effects of roof slope and kneeling posture on the peak activation of knee muscles using EMG data. The findings suggest that increased roof slope during shingle installation elevates the risk of knee musculoskeletal disorders among roofers due to amplified muscle loading. Eilertsen et al. [94] developed an unpowered lift assist device for drywall installation, with EMG analysis revealing a significant reduction in muscle activation. 

Moreover, EMG is used to investigate “ergonomics” by optimizing work processes and environments, monitoring fatigue, and minimizing physical strain. Brandt et al. [43] employed sEMG alongside other technical measurements to identify instances of excessive physical workload in construction work. Whereas the participatory ergonomics intervention did not reduce these excessive workload events, it notably decreased general fatigue and enhanced workers’ influence over tasks. Bangaru et al. [95] proposed a comprehensive system for continuous monitoring of construction workers’ fatigue levels. Using EMG to measure forearm muscle activity and motion data, this system surpasses traditional heart rate metrics in assessing fatigue. Age-related differences in physical strain among construction and healthcare workers were also discussed using EMG. It revealed that older workers, despite facing similar or higher occupational demands, exhibited distinct muscle activity patterns [39]. Additionally, the integration of EMG with “machine learning” techniques is evolving, where automated analysis could predict potential fatigue levels based on non-invasive monitoring methods like computer vision [55].

#### 3.6.2. EEG

**Figure 11:**
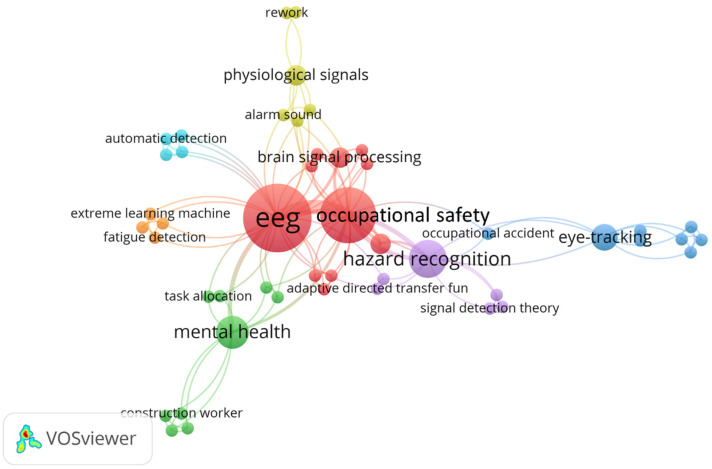
Keywords co-occurrence network of EEG in CHSM.

Figure 11 shows the co-word network of EEG in CHSM. Many studies have focused on the application of EEG in “occupational safety”, where it aids in identifying cognitive patterns that might indicate risks or inefficiencies in work processes. One particular aspect of interest has been “hazard recognition” in the construction environment. EEG has been instrumental in examining brain connectivity, revealing a top-down attention mechanism originating from the dorsal attention network [96]. From the psychology perspective, novice workers, armed with heightened sensitivity, often outperform their experienced counterparts due to more efficient working memory and attentional control [97]. Such neural responses, captured by EEG, are pivotal in understanding workers’ innate capabilities to identify hazards. 

Furthermore, EEG studies have also ventured into understanding “mental health” in CHSM. For example, heightened risk awareness in migrant construction workers, especially concerning environmental stressors like air and noise pollution, often correlated with improved health outcomes, though these patterns displayed variations based on factors like age and gender [98]. In another research, EEG pinpointed specific construction noises, such as those from saws and jackhammers, as significant triggers for negative emotional responses, underscoring the need to revisit noise regulations to prioritize human welfare [17]. Exploring the realm of stress, EEG, in conjunction with algorithms such as multi-class SVM and FCNN, adeptly identified varying stress levels among workers. Achieving an accuracy of 79.26%, this innovative approach holds promise for early stress detection and offers a potential pathway toward fostering a more supportive construction environment [16].

#### 3.6.3. Eye-Tracking

**Figure 12:**
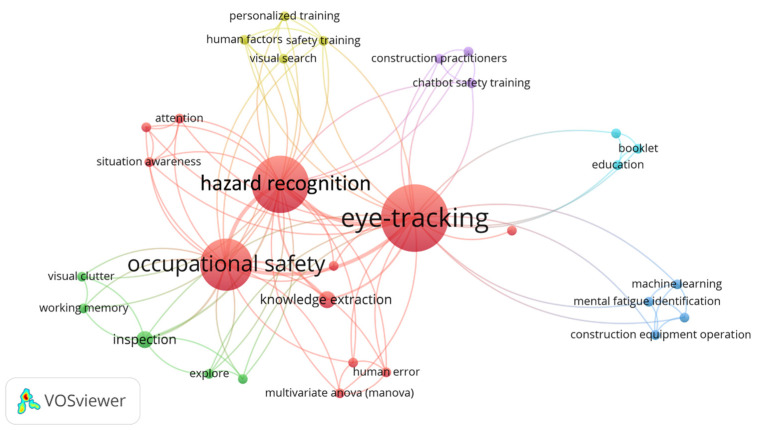
Keyword co-occurrence network of eye-tracking in CHSM.

Figure 12 demonstrates a co-word network of eye-tracking technology for construction applications. Eye-tracking has the capability to provide insights into visual attention and cognitive processes of workers in various scenarios. A predominant theme in CHSM research employing eye-tracking is “hazard recognition”. This tool uncovers how construction workers visually perceive, process, and respond to potential hazards, providing insights into their visual search patterns, focal points, and gaze durations. For instance, it was discovered that certain visual patterns, such as longer search durations and more expansive attention distribution, consistently correlated with superior hazard identification performance. Moreover, the integration of a personalized training intervention led to further refinement of these visual patterns, enhancing hazard recognition outcomes [57]. Another exploration into the technology illustrated the efficacy of systematic visual search strategies in the construction setting. By comparing the scan patterns of participants adept at hazard recognition with those less proficient, it is evident that focused, logical, and methodical visual searches were the symbol of effective hazard recognition [99]. In terms of “occupational safety and training”, Dzeng et al. [20] created a virtual environment to differentiate the visual search patterns of seasoned and novice workers during hazard identification. Experience was found to accelerate hazard assessment, yet it did not inherently improve the accuracy. However, the strategic and consistent search patterns of experienced workers indicate the promise of eye-tracking in personal safety training plans, ensuring enhanced hazard awareness for novices. Utilizing AI-based eye-tracking, the effectiveness of Telegram chatbot safety training in enhancing construction hazard awareness was assessed. The findings highlighted chatbot training’s role in boosting hazard recognition, suggesting the potential of affordable chatbot training as a key safety education tool [100]. Meanwhile, mobile eye-tracking revealed construction workers’ focus on various hazard sources, especially in areas with past fatal incidents. These observations illuminated the effectiveness of their prior safety training and emphasized the critical role of eye-tracking in shaping future safety measures [101].

#### 3.6.4. EDA

**Figure 13:**
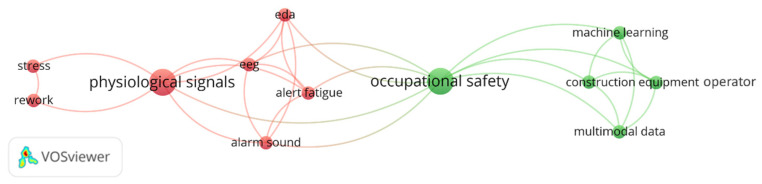
Keywords co-occurrence network of EDA in CHSM.

Figure 13 demonstrates the application of electrodermal activity in the construction field covariant word networks. A primary application of EDA in CHSM is toward understanding “occupational safety” in the part of stress response, alertness, and mental fatigue. By capturing the autonomic nervous system’s reactions through skin conductance, EDA provides invaluable insights into workers’ stress levels and emotional states. For instance, Chae and Kang [102] proposed an experimental design to gauge alert fatigue arising from different alarm sounds. This venture aimed to pinpoint the alarm sound that most effectively mitigated desensitization, especially in contexts like construction equipment blind spot scenarios. Meanwhile, when combined with EEG, EDA illustrated the pronounced stress responses in employees subjected to rework in engineering drawing tasks, highlighting the need for proactive stress management within the industry [66]. Mehmood et al. [44] combined EDA, EEG, and video signals in a multimodal fusion and demonstrated prowess in classifying mental fatigue during construction equipment operations. This approach garnered an impressive 96.2% accuracy with the decision tree model.

## 4. Discussion

This research identifies four key application areas for neuroscience tools in CHSM: monitoring safety status, improving hazard recognition ability, reducing work-related musculoskeletal disorders, and integrating with artificial intelligence to address the safety and health issues. Applications of these neuroscience tools introduced novel perspectives and methodologies to enhance the occupational safety and health of the construction industry, but there are inherent limitations persist in their practical applications in construction workplaces. This section discusses the constraints associated with the application of neuroscience tools in construction workplaces and highlights prospective research focus based on the main findings of the current research status.

### 4.1. Narrowing the Gaps between Experimental Settings and Real Situations

Most of the existing studies have used experimental studies, but the ecological validity of the experimental design needs to be improved in order to enhance the real-world applicability and generalizability of research outcomes. The experimental scenarios, being overly simplistic and not fully representative of the intricacies of construction sites, limited sample size, and different groups of subjects from the actual among existing studies, may make it to be less convincing to generalize the research findings in the real world. The reasons for these shortcomings mainly lie in that researchers would face many insurmountable challenges if completely replicating the real situation. Specifically, due to the complexity of the construction site, it is difficult to control experimental variables and to invite a large number of subjects to complete a long period of experimentation in the field [22]. For example, regarding the research on hazard identification ability, it would be impossible to ensure that the dynamically changing construction workplaces remain unchanged during the experimental period, and the subjects may be exposed to a hazardous environment. As a result, many existing researches have been conducted in the laboratory, e.g., asking subjects to identify hazards in a virtual construction site [103] or to detect hazards through viewing two-dimensional pictures on a computer screen [21,57]; however, these experimental settings still have gaps with the hazard recognition behaviors in the real world.

In addition to the limitations due to the experimental scenarios, the neuroscience tools also cause limitations to the experimental design. The results show that EEG has been widely applied for workers’ safety monitoring, but it would be quite difficult to implement these studies in job sites because the quality of EEG data is severely impacted by the movement of the human body and the interference from the environment. Thus, many studies have applied psychological paradigms to require subjects to perform on-screen experimental tasks [80,104,105]. Such experimental settings allow for high-quality EEG data but fail to reflect the actual dynamics of the situation.

In response to narrowing the gaps between experimental settings and the real situations, some studies have proposed the application of VR technology as a potential method, which could avoid exposing subjects to dangerous environments and provide subjects with a realistic experience [103,106]. However, it would be difficult for researchers to reproduce a completely realistic situation based on the current VR technology, where substantial investments in VR content development are required to geometrically model each observable element in the scenes. 

Furthermore, some existing studies also have limitations in their experimental subjects. Since experiments may require long periods of time and complex procedures, some studies have invited undergraduate students to replace construction workers (e.g., [104]), and it is hard to consider the individual differences among workers by inviting a large number of workers with various backgrounds (e.g., age, experience, and gender), which may result in bias and incompleteness of the findings. To conclude, future experimental designs need to consider the experimental scenario design and subject selection to improve the credibility and generalizability of the findings.

### 4.2. Enhancing the Quality of Data Collected by Neuroscience Tools and Performance of Data Processing Algorithms

Although neuroscience tools have been comprehensively explored to address key safety and health issues in the construction industry, the reliability of the research outcomes would also depend on the quality of the collected physiological data. Collecting data using these tools in construction sites is challenging because of the construction environment and the frequent movement of workers. Signals collected in the field are expected to be affected by a number of external signal artifacts, e.g., device power line interference, motion artifacts, and electrode motion artifacts [107]. In addition, the data are also affected by intrinsic signal artifacts, e.g., respiration, pulse, muscle, motion, and eye artifacts. These artifacts are common in any physiological sensing data (e.g., PPG, ECG, EDA) [107].

To address these issues, past studies have directed their efforts toward the processing of EEG data. For example, Jebelli et al. [108] developed a framework for processing intrinsic signal artifacts (e.g., blinks, vertical eye movements, and muscle movements) and extrinsic signal artifacts (e.g., sensor displacements, body motions, electrode line noise, etc.) collected from wearable EEG headsets. Wang et al. [109] proposed a new hybrid kinematic-EEG data type and adopted wavelet packet decomposition to compute the vigilance measurement indices with redefined EEG sub-bands, which could reduce the artifacts caused by muscle movements. The researchers have focused more on EEG data, possibly because these data have found to have great potential for indicating workers’ safety status [23]. However, there are great limitations in applying EEG in construction sites due to the stringent nature of EEG data collection. Some studies in recent years have hence attempted to explore peripheral physiological signals, such as ECG [110] and EDA [62], which are easier to collect in job sites to replace EEG to reflect cognitive status. With the wide application of other tools, future research is recommended to examine the quality of signals collected by other kinds of neuroscience tools under diverse conditions at construction sites to eliminate and reduce the defects in the acquired signals.

Moreover, applications of the neuroscience tools in job sites also depend on the data mining algorithms. The results show that monitoring the status of workers is a major application point, and the research is now leaning toward incorporating AI techniques to solve the problem. This is because a huge amount of data will be produced if deploying neuroscience tools on construction workers, and AI techniques, such as machine learning algorithms and deep learning algorithms, are advantageous tools for predicting workers’ state of safety and health from these large amounts of data. Due to the complex nature of construction sites with ever-changing conditions and diverse worker behaviors, these AI systems must constantly learn and adapt [111,112]. Future studies are suggested to investigate appropriate algorithms for the safety and health predictions. 

### 4.3. Overcoming User Resistance in Tool Adoption

Although existing research has shown the potential for the application of neuroscience tools to improve construction safety and health, it is difficult to fully benefit from these applications if construction workers refuse to adopt it. Choi et al. [113] investigated the factors influencing the intention of construction workers to adopt wristbands that monitor their heart rate during the workday. The results showed that perceived usefulness, perceived privacy risk, and social influence were common factors that influenced employees’ intention to accept the tool. There are still gaps in the knowledge from existing studies about which features of the tools (e.g., functionality and design) and the context of use influence the direct determinants of acceptance. Moreover, workers may even resist monitoring their mental states through the application of these wearable neuroscience tools. Interventions aimed at influencing or altering the cognitive or behavioral attributes of workers are ethically sensitive, with the potential for unintended effects and the infringement of individual freedoms. Therefore, the integration of neuroscience tools into health and safety management (CHSM) must be accompanied by a rigorous response to the intertwined ethical, moral, and legal challenges.

## 5. Conclusions

The integration of neuroscience tools into CHSM has manifested as a pivotal advancement in recent scientific development. This study undertook a comprehensive bibliometric analysis, encompassing an array of publications related to the application of neuroscience tools within the construction safety and health field. Initial descriptive analysis illustrates the foundational characteristics of the publications, elucidating prominent contributors in terms of countries, institutions, and authors. From a nascent stage with minimal publications in the early 2000s, the research related to CHSM based on neuroscience tools has witnessed a meteoric advance in the late 2000s, followed by a phase of stabilization in recent years, reflecting the maturation and sustained interest in the domain. Notably, journals such as *Automation in Construction*, *Journal of Construction Engineering Management*, and *Journal of Management Engineering* in the construction field are emerging as leading outlets for research dissemination in this niche. 

Co-occurrence analysis in keywords discerned four central topics within the publications: monitoring the safety status of construction workers, enhancing the construction hazard recognition ability, reducing work-related musculoskeletal disorders of construction workers, and integrating neuroscience tools with artificial intelligence techniques in enhancing occupational safety and health. Among these neuroscience tools, Magnetoencephalography (EMG), Electroencephalography (EEG), eye-tracking, and Electrodermal Activity (EDA) were identified as the four predominant neuroscience tools.

In conclusion, integrating neuroscience tools into construction health and safety management has transformed the research landscape over the past decades. This integration advanced through converging neuroscience with emerging computational techniques, enabling data-driven worker safety decisions. With technological innovations continuously propelling neuroscience tools forward and increased the prioritization of worker health and safety, research in this domain is poised for sustained growth and impact.

There are still some gaps in applying these tools in construction workplaces. Future studies are suggested to narrow the gaps between experimental settings and real situations, enhance the quality of data collected by neuroscience tools and performance of data processing algorithms, and overcome user resistance in tools adoption to facilitate the applications of these tools in construction workplaces.

Whereas this review has endeavored to offer a comprehensive examination of the existing publications in CHSM based on neuroscience tools, several limitations need to be acknowledged. The selection of specific keyword combinations based on a review of existing literature may have missed studies that used different terms. Moreover, the focus on English-language literature potentially introduces a regional bias, sidelining significant findings published in other languages. Additionally, while descriptive analysis, co-citation, and co-occurrence analysis offer valuable insights, they might not fully encapsulate emerging trends or intricate dynamics in the literature. Future research should consider a broader keyword spectrum, integrate multi-language studies, and employ advanced bibliometric methods to address these gaps. These refinements can provide more profound and global insights, ensuring a more holistic understanding of the field.

## Figures and Tables

**Table 1:** Search query.

Database	Search Query
Web of Science (*n* = 53) Scopus (*n* = 185) PubMed (*n* = 21)	(“construction industry” OR “construction sector” OR “construction organization” OR “building industry” OR “building project”) AND (“construction safe *” OR “worker safe *” OR “labor safe *” OR “accident perception” OR “collision perception” OR “risk recognition” OR “hazard recognition” OR “danger recognition” OR “vigilance” OR “attention” OR “alertness” OR “notice” OR “cognitive status” OR “cognitive state” OR “mental status” OR “mental state” OR “brain status” OR “brain state” OR “psychological status” OR “psychological state” OR “fatigue” OR “exhaustion” OR “work load” OR “mental load” OR “cognitive load” OR “stress” OR “tension” OR “emotion” OR “occupational health *” OR “physical strain” OR “ergonomic risk” OR “muscle fatigue” OR “musculoskeletal disorder” OR “spinal compression” OR “injury” OR “illness” OR “therapy” OR “interference” OR “recovery”) AND (“Electroencephalogram” OR “EEG” OR “eye tracking” OR “ET” OR “event related potential” OR “ERP” OR “Electrocardiogram” OR “ECG” OR “Electromyography” OR “electromyogram” OR “EMG” OR “Electrodermal activity” OR “EDA” OR “Photoplethysmography” OR “PPG” OR “functional near-infrared spectroscopy” OR “fNIRS” OR “functional magnetic resonance imaging” OR “fMRI” OR “magnetoencephalography” OR “MEG”)

Note: * represents a wildcard in the search formula to help search for more keywords.

**Table 2:** Total citations of institutions with more than two publications.

Institute	Number of Publications	Total Citations
City University of Hong Kong	4	295
Tsinghua University	4	55
EMGO+ Institute for Health and Care Research	3	128
Purdue University	3	43
University of Florida	3	31
Coronel Institute of Occupational Health	2	105
Huazhong University of Science and Technology	2	116
National Institute of Occupational Health	2	54
North Carolina State University	2	88
The Hong Kong Polytechnic University	2	91
University of Michigan	2	107
University of Nebraska-Lincoln	2	166
University of Waterloo	2	32

**Table 3:** Most influential author analysis.

Author	TC	NP	H-Index	Ave. Pub. Year	Affiliation	Research Subject
Li, Heng	273	5	4	2020	The Hong Kong Polytechnic University, Hong Kong SAR, China	Construction Informatics, Construction Engineering and Management, Construction Health and Safety
Berardi, Umberto	272	1	1	2012	Toronto Metropolitan University, Canada	Sustainability, Energy Efficiency, Acoustics, Urban Resiliency, Building Science
Chen, Jiayu	263	4	5	2017	City University of Hong Kong, Hong Kong SAR, China	Human-centric Mental Sensing, Human-Robot Collaboration, Building Digital Twin, Smart Urban Grid and Simulation
Esmaeili, Behzad	258	4	5	2018	Purdue University, United States	Construction Safety, Risk Management, Decision Making
Hasanzadeh, Sogand	257	4	4	2019	Purdue University, United States	Smart Safety, Wearable TechAR/VR/MR, Human-AI Teaming, Human Factor
Wang, Di	143	2	2	2018	University of Michigan, United States	Deep Learning, Signal Processing, Mutual Information
Choi, Byungjoo	137	3	3	2020	Ajou University, South Korea	Construction Engineering and Management, Construction Automation, ICT in Construction, Smart Construction, Construction Safety
Jebelli, Houtan	121	3	4	2020	Pennsylvania State University, United States	Construction Robotics, Construction Automation, Human Robot Collaboration, Wearable Technologies, Engineering Education
Van Der Molen, Henk F	118	3	3	2013	VU University Medical Center, Netherlands	Construction Worker, Musculoskeletal Disorders, Work-related Risk Factors, Preventive Measures, Physical and Mental Health
Dzeng, Ren-Jye	112	2	1	2016	National Chiao Tung University, Taiwan, China	Construction Management, Artificial Intelligence, Mobile App, Sensor, Eye tracker
Lin, Chin-Teng	111	1	1	2016	National Chiao Tung University, Taiwan, China	Computational Intelligence, Machine Learning, Fuzzy Neural Networks, Cognitive Neuro-Engineering, Brain-Computer Interface
Song, Xinyi	111	1	2	2016	Georgia Institute of Technology, United States	Building Energy Efficiency, Occupational Health, Facility Management, Construction Safety, Construction Dispute
Boschman, Julitta S.	110	2	2	2012	University of Amsterdam, Netherlands	Musculoskeletal Disorders, Occupational Health, Work-related Health Surveillance, Processing Assessment
Albert, Alex	92	3	3	2019	North Carolina State University, United States	Construction Safety, Injury Prevention, Risk Management, Hazard Recognition, Safety Interventions
Yu, Yantao	91	1	1	2019	The Hong Kong Polytechnic University, Hong Kong SAR, China	Construction Informatics, Wearable Device, Behavior Recognition

**Table 4:** Most cited documents.

Author	Title	Source	Year	TC	Research Focus
Dzeng R. J.; Lin C. T.; Fang Y. C. [20]	Using eye-tracker to compare search patterns between experienced and novice workers for site hazard identification	Safety Science	2016	111	Examining the impact of experience on hazard identification in digital construction sites using eye-tracking, highlighting the difference between speed and accuracy.
Chen J.; Song X.; Lin Z. [45]	Revealing the “invisible Gorilla” in construction: Estimating construction safety through mental workload assessment	Automation in Construction	2016	100	Examining the role of inattentional blindness on hazard identification in construction sites using eye-tracking and EEG analysis.
Wang D.; Chen J.; Zhao D.; Dai F.; Zheng C.; Wu X. [14]	Monitoring workers’ attention and vigilance in construction activities through a wireless and wearable electroencephalography system	Automation in Construction	2017	98	Exploring the effectiveness of a wearable EEG system in evaluating the attention of construction workers.
Boschman J.S.; Van Der Molen H.F.; Sluiter J.K.; Frings-Dresen M.H. [54]	Musculoskeletal disorders among construction workers: A one-year follow-up study	BMC Musculoskeletal Disorders	2012	97	Using questionnaires to assess the prevalence, work-relatedness, and work problems of musculoskeletal disorders among different construction occupations to examine the value of choosing preventive measures.
Hasanzadeh S.; Esmaeili B.; Dodd M.D. [21]	Measuring the impacts of safety knowledge on construction workers’ attentional allocation and hazard detection using remote eye-tracking technology	Journal of Management in Engineering	2017	95	Employing eye-tracking to examine the effects of safety knowledge on hazard detection among construction workers.
Yu Y.; Li H.; Yang X.; Kong L.; Luo X.; Wong A.Y.L. [55]	An automatic and non-invasive physical fatigue assessment method for construction workers	Automation in Construction	2019	91	Introducing a computer vision technique to assess construction workers’ physical fatigue through 3D motion capture and biomechanical analysis.
Hasanzadeh S.; Esmaeili B.; Dodd M.D. [22]	Examining the relationship between construction workers’ visual attention and situation awareness under fall and tripping hazard conditions: Using mobile eye tracking	Journal of Construction Engineering and Management	2018	87	Studying the relationship between construction workers’ attention and situational awareness using eye-tracking.
Jeelani I.; Albert A.; Han K.; Azevedo R. [57]	Are visual search patterns predictive of hazard recognition performance? Empirical investigation using eye-tracking technology	Journal of Construction Engineering and Management	2018	70	Using eye-tracking to analyze the link between construction workers’ visual patterns and hazard recognition performances and the impact of tailored training on enhancing these patterns and performance.
Jebelli H.; Choi B.; Lee S.H. [58]	Application of wearable biosensors to construction sites. I: Assessing workers’ stress	Journal of Construction Engineering and Management	2019	66	Creating a framework using wearable sensors and machine learning to continuously predict construction workers’ stress from physiological signals.
Umer W.; Li H.; Szeto G.P.Y.; Wong A.Y.L. [59]	Identification of biomechanical risk factors for the development of lower-back disorders during manual rebar tying	Journal of Construction Engineering and Management	2017	63	Analyzing lumbar biomechanics in various postures during rebar tying, highlighting stooping’s role in increased lower back disorders in rebar workers.

**Table 5:** Cluster terms.

Cluster	Keywords
1	occupational safety (0.28) EEG (0.22) wearable biosensor (0.09) physiological signals (0.06) activity recognition (0.03) aerobic fatigue threshold (0.03) aging (0.03) attention (0.03) construction labor shortage (0.03) fatigue monitoring (0.03) muscle activity (0.03) oxygen prediction (0.03) scaffold building (0.03) inattentional blindness (0.03) occupational stress (0.03) supervised learning (0.03) brain signal processing (0.02) construction worker physical demand (0.02) construction workers’ stress prediction (0.02) performance (0.02) alarm sound (0.02) alert fatigue (0.02) cognitive ability (0.02) construction activity classification (0.02) data quality (0.02)
2	eye-tracking (0.18) hazard recognition (0.16) risk management (0.14) mental health (0.10) virtual reality (0.06) safety training (0.04) working memory (0.04) occupational accident (0.04) aviation (0.04) cognitive load (0.04) labor and personnel issues (0.04) maritime (0.04) training (0.04) visual attention (0.04) decision dynamics (0.03) mixed reality (0.03) age (0.03) disabilities (0.03) experience (0.03) individual characteristics (0.03) inspection (0.03) sleep disorders (0.03)
3	occupational health (0.13) musculoskeletal disorders (0.13) construction worker (0.11) EMG (0.10) low back pain (0.07) ergonomic (0.06) shoulder pain (0.06) back pain (0.04) endurance (0.04) fatigue (0.04) modelling (0.04) recovery (0.04) participatory ergonomics (0.04) action research (0.04) economics (0.04) fatigue reactions (0.04) heavy industries (0.04) musculoskeletal pain (0.04) organizational ergonomics (0.04) postural stability (0.04) construction noise (0.03) environmental pollution (0.03) dietary behaviour (0.03) energy balance related behaviour (0.03)
4	construction industry (0.31) machine learning (0.08) time pressure (0.05) prevention (0.04) deep learning (0.04) computer vision (0.04) mental fatigue (0.03) panel data (0.03) construction equipment operator (0.03) noise-induced hearing loss (0.02) occupational hearing conservation (0.02) screening (0.02) self-administered (0.02) speech reception threshold (0.02) speech-in-noise (0.02) accident (0.02) disability (0.02) handicap (0.02) involvement (0.02) lean (0.02) matrix (0.02) workforce (0.02) correlation and influence (0.02) disaggregation (0.02) emotional exhaustion (0.02)
